# Comparison: real and simulated ear insertion gain

**DOI:** 10.1590/S1808-86942011000500003

**Published:** 2015-10-22

**Authors:** Patrícia Danieli Campos, Maria Fernanda Capoani Garcia Mondelli, Debora Viviane Ferrari

**Affiliations:** 1 Specialist in clinical and educational audiology, Federal Board of Speech Therapy. Laboratory specialist on hearing aids, Speech Therapy Department, Bauru Dentistry School, São Paulo University; 2 Doctoral degree in communication disorders, HRAC, São Paulo University. Assistant professor of the speech therapy course, Bauru Dentistry School, São Paulo University; 3 Doctoral degree in neuroscience and neuropsychology, São Paulo University. Assistant professor of the speech therapy course, Bauru Dentistry School, São Paulo University. Bauru Dentistry School, São Paulo University

**Keywords:** hearing aids, hearing loss, simulation

## Abstract

**Abstract:**

The development of hearing aid (HA) software programming does not replace the analysis of real ears with probe microphones.

**Aim:**

To compare simulated insertion gain in HA software programming and real ear insertion gain.

**Method:**

A prospective study of 62 patients (aged from 29 to 93 years; 30 male and 32 female). All patients presented unilateral (n=14) or bilateral (n=48) and mild to profound sensorineural hearing impairment. 110 ears assessed. Data was gathered from medical records and the insertion gain was obtained in real ears for comparison with the simulated insertion gain in HA software programming. Statistical tests were applied to analyze the correlation of data - difference of real ear and simulated insertion gain.

**Results:**

HA software programming simulated insertion gain was higher than real ear insertion gain obtained with probe microphone measures. There were statistically significant frequency differences. Age did not correlate with the difference of real ear and simulated measures.

**Conclusion:**

The use of real ear measures is important during verification of HA.

## INTRODUCTION

A probe microphone measurement is an objective and accurate technique to check whether hearing aid (HA) performance in the user's ear fits into a certain curve or set or curves (gain or output per prescribed frequency); it is considered best practice for hearing aid fitting^1^, ^2^, ^3^, ^4^, ^5^, ^6^, ^7^. There are reports stating that about 23%-40% of professionals routinely use such measurements in clinical practice^8^, ^9^, ^10^.

Use of digital HAs has been accompanied by a growing trust by professionals in programming software simulations and calculations, rather than empirical verification of HA fit^11^.

Such software have become more sophisticated and at the same time user friendly, providing clinicians with interactive programming procedures, electronic fine tuning guides, and several visualization options as to how a given HA is being programmed - such as simulated gain curves and output^1^,^12^. However, even if software demonstrates an equivalence between HAs and prescriptions, these simulations do not truly reflect the performance of these devices in the user's ear. Studies have shown that software simulations tend to overestimate the amplification that is actually provided to the real ear^12^, ^13^, ^14^.

The purpose of this study was to compare HA software programming simulated insertion gain with true insertion gain measurements.

## METHOD

The institutional review board approved this study (no. 28/2009).

A retrospective study was made of the registries of patients seen from January to July 2009; enrolment was done according to the following criteria:

- age group: adults (aged over 18 years),
- no outer or middle ear alterations as seen in the otorhinolaryngological examination,
- sensorineural hearing loss of varied degrees,
- users of software programmable HA with the NOAH 3.0 application,
- programming software with a simulated insertion gain option using the same parameters as those used in real ear measurements (speech spectrum stimulus, and presentation level at 65 dB SPL),
- HA evaluated with the noise reduction algorithm turned off.

Data was gathered from the registries of 62 patients (30 male and 32 female) aged from 29 to 93 years (mean - 71 years) with mild to severe unilateral (n=14) or bilateral (n=48) sensorineural hearing loss. The total number of ears was 110.

Table 1 shows the number of patients according to age groups (10-year intervals).

**Table 1:** Distribution of patients according to age groups.

Age group	Number of patients
21 30	1
41 50	2
51 60	8
61 70	19
71 80	15
81 90	16
91 100	1

Probe microphone measurements were done in acoustic booths that were large enough for patients to be seated one meter away from the loudspeaker at 0**+** azimuth. The examiner used the geometric method to position the probe tube for in situ measurements. Thus, the probe tube was aligned along the medial ear mold position and measured so that it remained 5.0 mm beyond the tip of the patient's ear mold and 5.0 to 6.0 mm from the tympanic membrane. A marker ring was placed on the intertragic notch to check the probe tube length to be inserted in the outer ear canal of the patient. A speech noise signal at 65 dB SPL was used for the measurements. An Aurical (Madsen) device was used for results analysis.

The database of the NOAH 3.0 platform was used to gather data on software programming simulated insertion gain from two HA providing companies, which presented simulation data as simulated insertion gain charts per frequency generated by a speech noise stimulus at 65 dB.

The software simulation results and real ear measurements at 250 to 4000 Hz interoctave bands were recovered and annotated in a specific protocol.

The statistical analysis was made with the *Pacote Estatístico (PACOTICO)* software. The paired t test was applied to compare the true insertion gain with the simulated insertion gain at each test frequency. Pearson's correlation coefficient was applied to check for correlations between age and the difference between software simulated and real ear values. The significance level (α) was = 0.05 in all cases.

## RESULTS

**Figure 1:**
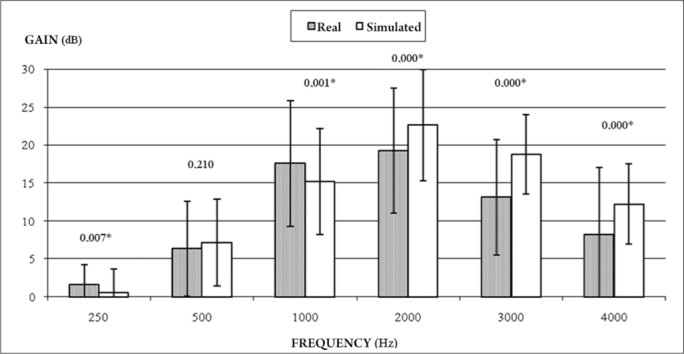
Comparison between true gain obtained from measurements with a microfone sonda and simulated gain in the hearing aid programming software at 250-4000 Hz (n=110).

Figure 1 presents the true and simulated gains per frequency and their significance.

Table 2 shows the correlations among age and differences between simulated and true gains and their significance.

**Table 2:** Correlation (Pearson) between the age of participants and the mean difference at different frequencies.

Frequency (Hz)	Correlation (r)	(*p*)
250Hz	-0,11	0,26
500Hz	-0,16	0,09
1000Hz	-0,06	0,55
2000Hz	-0,10	0,31
3000Hz	-0,16	0,10
4000Hz	-0,15	0,11

## DISCUSSION

Figure 1 shows that there was a significant difference between simulated and real insertion gains at all frequencies except 500 Hz; such differences were larger at higher frequencies.

Differences between true and simulated gains have been noted in the literature^12^. A study of 12 patients showed that differences between true and simulated gains were 0 dB (± 5 dB standard deviation) from 250 to 1000 Hz. However, at higher frequencies, more patients showed substantial decreases in true insertion gains compared to simulated gains. Furthermore, negative difference values for most patients indicated that simulated insertion gains overestimated the true insertion gains in real ears. Such data agree with the findings of the present study, where the largest differences were encountered at 2000 to 4000 Hz. The values of real ear insertion gains were higher compared to simulated insertion gains at 250 to 1000 Hz; we found no reports of such inversion of differences in the literature.

A few authors^13^ have stated that real ear measurements simulated in HA programming software are not accurate, and tend to overestimate individually obtained values. These values were 0.5 dB lower at 1000 Hz and over 4000 Hz. True insertion gain values are closer to simulated insertion gain values at frequencies from 1500 to 4000 Hz. Our data revealed differences even at frequencies from 2000 to 4000 Hz; we were unable to assess frequencies over 4000 Hz because of limitations in the software charts of companies supplying HAs.

Results have shown that *64%* of fits did not reach the prescribed insertion gain by the NAL-NL1 rule at one or more frequencies from 250 to 4000 Hz when the first fit or quick set option was chosen in the programming software^14^.

Correlations between age and simulated and true gain were weak and not significant (Table 2). We assume these results are due to a homogeneous study sample, since most subjects were aged from 70 to 90 years (Table 1).

Thus the importance of including probe microphone measurements according to current protocols in the verification process, making HA fitting a more objective process based on scientific evidence. Programming software are essential tools that provide interactivity with technology and enable more accurate adjustments. However, it is important to evaluate the meaning of each simulation chart and to interpret them as a starting point in HA fitting - a preadjustment with posterior verification should be considered.

Professionals should learn how companies providing these devices have generated the simulation values, which may be used as a first step.

## CONCLUSION

This study enabled us to assess differences between simulated and true (real ear) insertion gain values.

No correlation was found between age of participants and the differences between simulated and true insertion gain values; there were, however, a difference between true and simulated gain.
